# Fatal fulminant septic shock with disseminated intravascular coagulation following percutaneous aspiration and sclerotherapy of a hepatic cyst: a case report

**DOI:** 10.3389/fmed.2026.1845287

**Published:** 2026-06-17

**Authors:** Rong Deng, Yanlong Li, Jiale Chen, Yexuan Wang, Wenmin Chai, Danyun Zuo, Yingzong Lan

**Affiliations:** 1 College of Integrated Traditional Chinese and Western Medicine, Gansu University of Chinese Medicine, Lanzhou, China; 2 Department of Spleen and Stomach Diseases, Gansu Provincial Hospital of Traditional Chinese Medicine, Lanzhou, China

**Keywords:** disseminated intravascular coagulation, Escherichia coli, liver cyst aspiration, percutaneous aspiration, septic shock

## Abstract

Liver cysts are common benign lesions. Although percutaneous aspiration combined with sclerotherapy is a safe and effective treatment, severe infectious complications are rare but potentially fatal. This report describes a rare case of a 69-year-old female patient who underwent percutaneous aspiration and polidocanol sclerotherapy for multiple liver cysts. Postoperatively, she rapidly developed fulminant septic shock complicated by disseminated intravascular coagulation (DIC), ultimately resulting in death. Laboratory tests following onset of symptoms revealed significantly elevated inflammatory markers and coagulation disorders. Despite aggressive treatment with broad-spectrum antibiotics, fluid resuscitation, vasoactive agents, anticoagulation therapy, and continuous renal replacement therapy, the patient died within 24 h of the onset of shock. Escherichia coli was detected in both blood and cyst fluid cultures, suggesting a combined pathogenic mechanism involving both percutaneous puncture-related dissemination and biliary-intestinal bacterial translocation. This case demonstrates that percutaneous interventional treatment of liver cysts may lead to complications of fulminant infection, underscoring the critical importance of early recognition and timely intervention.

## Introduction

Liver cysts are common benign lesions; asymptomatic liver cysts do not require treatment. As liver cysts grow and exert pressure, they can cause abdominal discomfort, pain, bloating, and dietary symptoms such as nausea, vomiting, a sensation of fullness, and early satiety. Percutaneous aspiration combined with sclerotherapy has become the standard minimally invasive treatment for symptomatic liver cysts (1). Although this procedure is generally safe, rare and serious complications, such as infection, may still occur (2). Currently, there are very few reported cases of postoperative disseminated intravascular coagulation (DIC)leading to fulminant septic shock. This article reports a fatal case and discusses its potential pathogenesis and clinical implications.

## Case presentation

**Figure 1:**
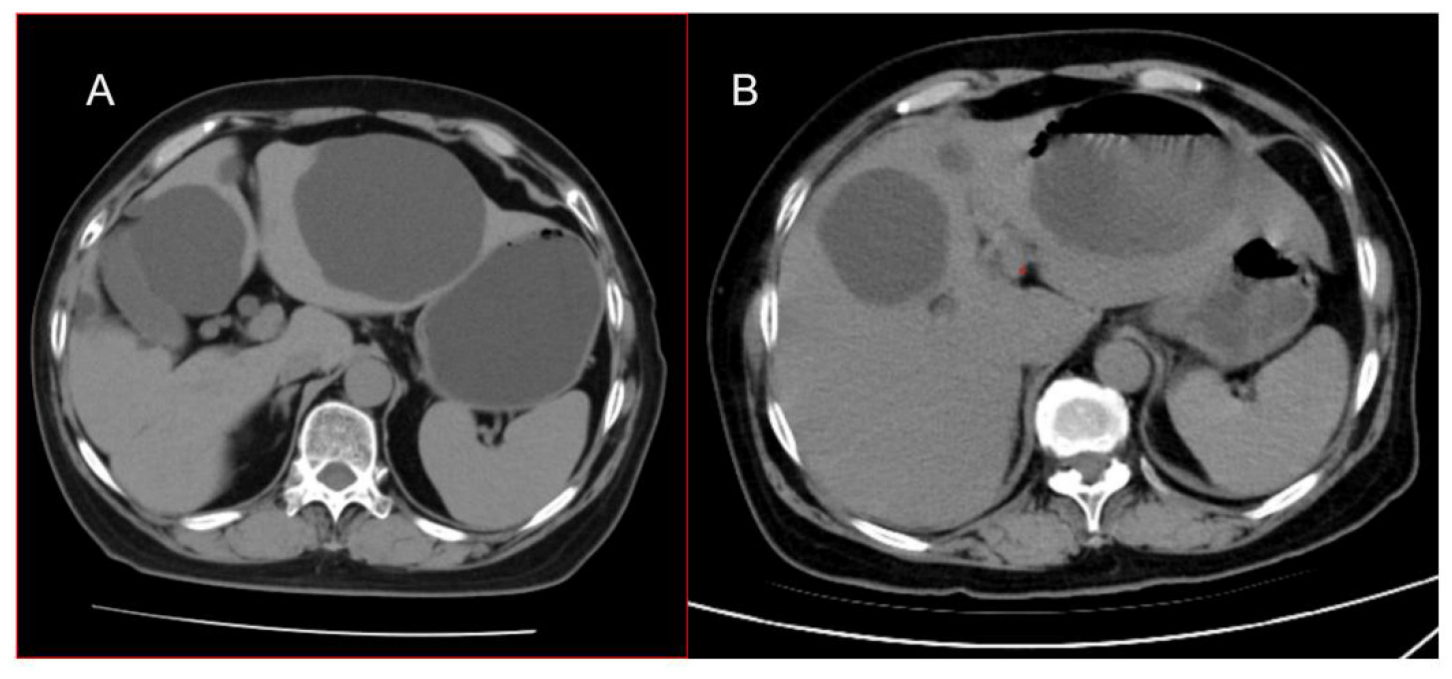
CT Imaging of Preoperative and Postoperative Sepsis in Patients with Liver Cysts, **(A)** Before a liver cyst aspiration, **(B)** After sclerotherapy for a liver cyst.

Patient is a 69-year-old female, urban resident, with a history of liver cysts for more than 18 months. On September 23, 2024, an ultrasound at another hospital indicated multiple liver cysts, the largest measuring 83^*^71 mm. On January 25, 2026, a follow-up abdominal CT during physical examination showed multiple cystic lesions in the left lobe of the liver, the largest about 90^*^70 mm, with clear borders and smooth cyst walls, with no obvious signs of infection (such as wall thickening or septation), (See Figure 1a). The preliminary diagnosis was multiple liver cysts. On March 5, 2026, at 3:00 p.m., the patient underwent ultrasound-guided percutaneous aspiration and sclerotherapy of a hepatic cyst. Following local anesthesia, a needle was inserted into the cyst cavity, and approximately 330 mL of pale yellow, clear cyst fluid was aspirated. Subsequently, 30 mL of 1% polidocanol (approximately 9% of the aspirated fluid volume) was slowly injected into the cyst cavity for sclerotherapy. The solution was left in place for approximately 15 min, and the patient's position was periodically adjusted to ensure adequate contact between the sclerosing agent and the cyst wall. Finally, the remaining solution in the cyst was aspirated as completely as possible. The procedure was uneventful, and no immediate complications were observed during the procedure. Approximately 26 h postoperatively, the patient suddenly experienced dizziness, nausea, and vomiting; the vomitus consisted of gastric contents. The patient's temperature was 37.8 °C. Following a complete blood count and C-reactive protein (CRP) test, the patient was treated with ceftriaxone, after which the temperature returned to normal. Forty hours postoperatively, the patient's condition rapidly deteriorated. She developed a sudden high fever (39.0 °C), accompanied by chills and upper abdominal pain and discomfort. She also exhibited hypotension, tachycardia, and somnolence, along with mottled skin changes. She was immediately transferred to the intensive care unit, where she was diagnosed with severe sepsis and disseminated intravascular coagulation (See Table 1). The assessment of disease severity yielded the following results: sequential organ failure assessment (SOFA) score of 12, APACHE II score of 32, international society on thrombosis and haemostasis (ISTH) DIC score of 8, and an in-hospital predicted mortality rate of 90%. Laboratory tests revealed an elevated white blood cell count, significantly elevated C-reactive protein and procalcitonin levels, and elevated blood lactate levels. A repeat CT scan showed a new air-fluid level within the cyst in the left hepatic lobe compared to the pre-puncture CT scan (See Figure 1b). Following diagnosis, comprehensive critical care measures were immediately initiated in accordance with sepsis treatment guidelines, including broad-spectrum antibiotics, fluid resuscitation, vasoactive agents, anticoagulation therapy, and continuous renal replacement therapy. However, the patient progressed to refractory septic shock and died of septic shock 24 h after the onset of symptoms. Despite mechanical ventilation and intensive care support, the patient died approximately 24 h after the onset of septic shock. Final blood culture and cyst fluid culture results both indicated an Escherichia coli infection, confirming the liver cyst as the source of infection.

**Table 1:** Postoperative laboratory tests.

Date and Time	WBC (× 10^9^/L)	CRP (mg/L)	PLT (× 10^9^/L)	APTT (s)	PTNR ratio	FIB (g/L)	dimer (mg/L)	FDP (ug/ml)
2026.3.4 11:06:10	3.85	< 6	147	27.8	0.96	2.17	0.15	–
2026.3.6 20:59:54	7.64	160.44	136	–	–	–	–	–
2026.3.7 11:15:29	–	–	–	43.8	1.51	3.41	66.18	
2026.3.7 12:22:08	9.83	222.49	52	–	–	–	–	–
2026.3.7 17:11:58	13.27	238.17	35	46.2	1.56	4.38	70.24	122.88
2026.3.7 12:22:08	10.71	305.91	8	133.1	1.76	4.38	41.98	90.64

Reference Ranges for Laboratory Tests: WBC (3.5–9.5 × 10^9^/L), CRP (0–6 mg/L), PLT (125–350 × 10^9^/L), APTT (25.4–38.4 s), PTNR (0.8–1.2), FIB (2.38–4.98 g/L), D-dimer (0–0.5 mg/L), FDP (0–2.01 ug/ml).

## Discussion

In this case, the onset of infection may involve the combined action of multiple factors, with the underlying mechanisms primarily including infection spread via biliary-related pathways and intervention-related factors. Previous studies suggest that, under specific conditions, some cystic liver lesions may have occult communication with the biliary system; particularly when there is chronic inflammation or structural changes in the cyst wall, enteric bacteria may enter the cyst cavity via bile reflux, thereby creating a potential basis for infection (3, 4). Furthermore, elevated biliary tract pressure and dysfunction of the sphincter of Oddi may also increase the risk of ascending bacterial infection (5). However, preoperative imaging in this case did not reveal clear signs of biliary communication, and intraoperative testing of cyst fluid bilirubin levels or contrast-enhanced imaging was not routinely performed; therefore, the possibility of minor or occult biliary communication cannot be completely ruled out. Escherichia coli was detected in both postoperative cyst fluid and blood cultures, suggesting that the source of infection is more likely to be enteric Gram-negative bacteria. Previous studies have confirmed that, in cases of impaired intestinal barrier function or alterations in the hepatic immune microenvironment, gut microbiota can translocate via the portal venous system or the biliary tract, thereby inducing systemic infection (6, 7).

Furthermore, percutaneous puncture itself may act as an independent risk factor through a mechanical dissemination effect, facilitating infection. Studies indicate that during invasive procedures, the needle tract can serve as a conduit for microbial migration, particularly when strict aseptic technique or prophylactic antibiotics are not adequately applied. Skin- or environment-derived bacteria may thus be introduced into deep tissue (8). Negative-pressure aspiration may additionally alter local pressure gradients, promoting bacterial translocation from the lesion into the bloodstream, thereby inducing procedure-related bacteremia. Hepatic cysts, as closed, nutrient-rich cavities, provide an ideal environment for rapid bacterial proliferation, and their limited blood supply restricts antibiotic penetration. Once bacteria enter the cyst cavity, high bacterial loads can be rapidly achieved, a phenomenon that may be referred to as the cystic amplification effect (9). Moreover, injection of sclerosing agents may cause local epithelial injury or necrosis, further enhancing conditions for bacterial growth.

On the basis of high bacterial burden, lipopolysaccharide (LPS) derived from Escherichia coli can activate Toll-like receptor 4 (TLR4) signaling, inducing the monocyte–macrophage system to release large amounts of pro-inflammatory cytokines, including TNF-α, IL-6, and IL-1β. This cascade triggers systemic inflammatory response syndrome and may escalate to a cytokine storm. Such a process not only causes widespread endothelial injury and increased capillary permeability but also directly drives coagulation system activation through the inflammation–coagulation interplay, representing a critical link between infection and coagulopathy (10).

This case further highlights that, in patients with large liver cysts, those adjacent to the biliary tract, or those with atypical imaging features, clinicians should be particularly vigilant for potential infection and biliary communication. Although fluoroscopic contrast evaluation has traditionally been used during percutaneous intervention to assess biliary communication, subtle or occult communication may still be underestimated using fluoroscopy alone. In this context, advanced image-guided techniques such as interventional radiology computed tomography (IVR-CT) or cone-beam CT may improve the detection of occult biliary communication and potentially enhance procedural safety in selected high-risk patients. Where necessary, cytological, biochemical (including bilirubin) and microbiological analyses of the cyst fluid should be performed to enhance the ability to identify risks. From a therapeutic perspective, the management principles for infectious and non-infectious liver cysts should be strictly distinguished. For cases where infection cannot be completely ruled out, it is recommended to prioritize antimicrobial therapy combined with adequate drainage; sclerotherapy should only be considered after the infection has been controlled, in order to reduce the risk of severe infection. Although current guidelines do not explicitly recommend the routine use of prophylactic antibiotics following simple liver cyst aspiration, individualized perioperative antimicrobial strategies for high-risk patients warrant further investigation (11).

## Conclusion

This case demonstrates that although percutaneous aspiration and sclerotherapy for hepatic cysts are generally safe, they may rarely lead to fulminant and fatal infectious complications. Occult infection or biliary communication should be considered in patients with large or atypical cysts. Routine cyst fluid analysis, careful preoperative risk assessment, and staged management when infection cannot be excluded may help reduce the risk of severe postoperative septic complications. Further studies are needed to clarify the role of prophylactic antibiotics in high-risk patients.

## Data Availability

The original contributions presented in the study are included in the article/Supplementary material, further inquiries can be directed to the corresponding author.
